# Circulating miRNA Expression Profiles and Machine Learning Models in Association with Response to Irinotecan-Based Treatment in Metastatic Colorectal Cancer

**DOI:** 10.3390/ijms24010046

**Published:** 2022-12-20

**Authors:** Evangelia Pliakou, Dimitra Ioanna Lampropoulou, Nikolas Dovrolis, Dimosthenis Chrysikos, Dimitrios Filippou, Christos Papadimitriou, Antonios Vezakis, Gerasimos Aravantinos, Maria Gazouli

**Affiliations:** 1 Second Department of Medical Oncology, General Oncology Hospital of Kifissia “Agioi Anargiroi”, Nea Kifissia, 14564 Athens, Greece; 2 ECONCARE, 11528 Athens, Greece; 3 Laboratory of Biology, Department of Medicine, Democritus University of Thrace, 68100 Alexandroupolis, Greece; 4 1st Department of Propaedeutic Surgery, Hippoctation Hospital, Medical School, National and Kapodistrian University of Athens, 11528 Athens, Greece; 5 Department of Anatomy, Medical School, National and Kapodistrian University of Athens, 11527 Athens, Greece; 6 Second Department of Surgery, Aretaieion Hospital, Medical School, National and Kapodistrian University of Athens, 11528 Athens, Greece; 7 Department of Surgery, Aretaieion University Hospital, Medical School, National and Kapodistrian University of Athens, 11528 Athens, Greece; 8 Department of Basic Medical Sciences, Laboratory of Biology, Medical School, National and Kapodistrian University of Athens, 11527 Athens, Greece

**Keywords:** microRNAs, colorectal cancer, machine learning, artificial intelligence, irinotecan, resistance

## Abstract

Colorectal cancer represents a leading cause of cancer-related morbidity and mortality. Despite improvements, chemotherapy remains the backbone of colorectal cancer treatment. The aim of this study is to investigate the variation of circulating microRNA expression profiles and the response to irinotecan-based treatment in metastatic colorectal cancer and to identify relevant target genes and molecular functions. Serum samples from 95 metastatic colorectal cancer patients were analyzed. The microRNA expression was tested with a NucleoSpin miRNA kit (Machnery-Nagel, Germany), and a machine learning approach was subsequently applied for microRNA profiling. The top 10 upregulated microRNAs in the non-responders group were hsa-miR-181b-5p, hsa-miR-10b-5p, hsa-let-7f-5p, hsa-miR-181a-5p, hsa-miR-181d-5p, hsa-miR-301a-3p, hsa-miR-92a-3p, hsa-miR-155-5p, hsa-miR-30c-5p, and hsa-let-7i-5p. Similarly, the top 10 downregulated microRNAs were hsa-let-7d-5p, hsa-let-7c-5p, hsa-miR-215-5p, hsa-miR-143-3p, hsa-let-7a-5p, hsa-miR-10a-5p, hsa-miR-142-5p, hsa-miR-148a-3p, hsa-miR-122-5p, and hsa-miR-17-5p. The upregulation of microRNAs in the miR-181 family and the downregulation of those in the let-7 family appear to be mostly involved with non-responsiveness to irinotecan-based treatment.

## 1. Introduction

According to the latest data, colorectal cancer (CRC) ranks third among the most common diagnosed malignancies worldwide [1]. De novo metastatic CRC (mCRC) has been linked with poorer prognosis compared to metachronous occurrences of metastases [2]. However, despite the discovery and addition of novel therapeutic approaches, treatment failure and drug resistance remain major obstacles in the battle against mCRC. The backbone of the regimens that are traditionally used for the treatment of mCRC are fluoropyrimidine-based schemes, including irinotecan and oxaliplatin. More specifically, these are the combinations that are more frequently used in day-to-day clinical practice: FOLFIRI (5-fluorouracil, leucovorin and irinotecan), CAPIRI (capecitabine and irinotecan), FOLFOX (5-fluorouracil, leucovorin and oxaliplatin), CAPOX (capecitabine and oxaliplatin) and FOLFOXIRI (5-FU, leucovorin, oxaliplatin and irinotecan). Additionally, targeted therapies such as anti-vascular epithelial growth factor (anti-VEGF) and anti-epidermal growth factor receptor (anti-EGFR) agents have further improved Response Rates (RR), Progression Free Survival (PFS) and Overall Survival (OS) in patients suffering from mCRC [3].

MicroRNAs (miRNAs) represent a non-coding RNA subtype that inhibits gene expression on a post-transcriptional level. Several remarkably stable forms of circulating miRNAs exist and can be isolated from body fluids and other extracellular environments such as blood, urine and saliva. Despite the fact that miRNA biogenesis is a highly controlled process, miRNA dysregulation has been directly associated with tumorigenesis and multiple cancer types, including CRC [4]. The differential expression of miRNAs has also been correlated with the molecular profile of CRC, tumor size and location (left versus right colon), presence of microsatellite instability (MSI) and response to cancer therapy [5]. Furthermore, their role in cancer drug resistance/response has been widely studied in several malignancies, including CRC [6,7,8]. Interestingly, alterations in the tumor microenvironment that result from the cell-to-cell transfer of miRNAs have been reported as one of the major causes of irinotecan resistance in human CRC [9]. Kannathasan et al. found that specific miRNA expression profiles have been associated with the activation of the G protein nucleolar 3-like (GNL3L)/NF-κB pathway [10]. Similarly, hsa-miR-155 was significantly associated with irinotecan resistance via targeting differentially expressed genes such as GPT2, NOB1 and KRCC1 [11]. Therefore, the current research supports the idea that circulating miRNAs are attractive candidates for the detection of novel drug resistance/response biomarkers, promising a wide spectrum of clinical applications in precision oncology.

Moreover, the growing progress of the field and the increasing amount of scientific and clinical data in experimental biology that have entered the mainstream of clinical practice warrant new molecular tools for individualized treatments. However, the unprecedented volume of large-scale miRNA expression profiling involves potential pitfalls in terms of extracting clinically significant results. To overcome this challenge, bioinformatics were developed and applied; this discipline basically combines computer technology tools in order to accurately capture, analyze and interpret massive and complicated sets of biological data and information [12]. Machine learning (ML) refers to a sub-category of artificial intelligence (AI) which uses computer algorithms in order to populate predictive models that derive from training data, while also being able to repeatedly self-adapt in order to improve its performance. Currently, machine learning has gained extensive scientific interest in oncology research and practice [13], and there is evidence that ML methods have been linked to favorable predictive patterns, especially when dealing with extensive data sets and large numbers of input variables. Indeed, classical statistical approaches may be affected by the complexity of the extended data, whereas ML may offer more suitable options, allowing for the analysis of large sets of data of RNA and microRNA sequencing. Additionally, ML combined with gene-ontology analysis provides valuable biological insights that are associated with the phenotype under investigation [14,15].

Acquired resistance is directly associated with cancer types as well as the administered regimens. Thus, the specific resistance mechanism may be unique, depending on the individual, the cancer subtype and the applied treatment. Unfortunately, until now, there were no tools that could accurately predict drug responses and support more of a precision oncology approach. However, as mentioned before, technological advances combining ML and biomedicine have been a promising field for the early cancer detection and treatment optimization. Indeed, several studies have investigated the role of ML in cancer drug response [16,17,18,19]. Moreover, despite the fact that several predictive models for both monotherapy and combination therapy have been developed, clinically relevant predictive models are still challenging to make significant in everyday clinical practice [20].

In light of the above, in the present work, we aimed to elucidate the expression profiles of circulating miRNAs, in association with the response to irinotecan-based treatment, in a cohort study including 95 Caucasian patients with mCRC. Moreover, we used ML approaches to identify the miRNAs that could serve as predictors of the response to irinotecan-based therapy in mCRC and offer an initial evaluation for future precision medicine applications in the clinical setting. Finally, we identified key molecular pathways relating to target genes through gene ontology analysis in order to better understand the mechanisms involved between the differential expression of circulating miRNAs and the response to irinotecan-based treatment.

## 2. Results

### 2.1. miRNA Differential Expression

**Figure 1:**
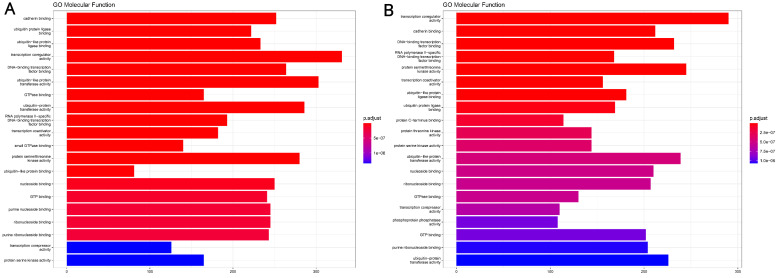
(**A**) Top 20 GO:MF terms enriched by our top 10 upregulated miRNAs; (**B**) Top 20 GO:MF terms enriched by our top 10 downregulated miRNAs.

Out of the 84 miRNAs of our array, 35 in total showed an expression dysregulation of statistical importance, with an adjusted -*p* < 0.05 and fold regulation of ±2 (Table 1). Of those, 17 were upregulated and 18 were downregulated in non-responders. Upregulation of miRNAs in the family miR-181 and downregulation of those in the let-7 family appear to be mostly involved with non-responsiveness to therapy, along with others. The miRNA hsa-let-7f-5p appears to be contrary to this trend. The top 10 upregulated miRNAs (hsa-miR-181b-5p, hsa-miR-10b-5p, hsa-let-7f-5p, hsa-miR-181a-5p, hsa-miR-181d-5p, hsa-miR-301a-3p, hsa-miR-92a-3p, hsa-miR-155-5p, hsa-miR-30c-5p, and hsa-let-7i-5p) were used as input for the identification of their target genes and the subsequent investigation of the pathways those enrich. In total, 9517 unique genes were identified, enriching 258 GO:MF terms (Supplementary go_enrich_up). In a similar fashion, the top 10 downregulated miRNAS (hsa-let-7d-5p, hsa-let-7c-5p, hsa-miR-215-5p, hsa-miR-143-3p, hsa-let-7a-5p, hsa-miR-10a-5p, hsa-miR-142-5p, hsa-miR-148a-3p, hsa-miR-122-5p, and hsa-miR-17-5p) identified 8676 unique genes which enriched 197 GO:MF terms (Supplementary go_enrich_down). Figure 1 depicts the top 20 GO:MF terms enriched by the up- and downregulated miRNAs of our analysis. This analysis highlights biologically important/tumor-related molecular functions like “cadherin binding,” “transcription coregulator activity,” “ubiquitin protein ligase binding,” “ubiquitin-like ligase binding,” “transcription coactivator activity,” “protein serine/threonine kinase activity,” “DNA-binding transcription factor binding” and “RNA polymerase II-specific DNA-binding transcription factor binding.”

### 2.2. miRNA Machine Learning Model

**Figure 2:**
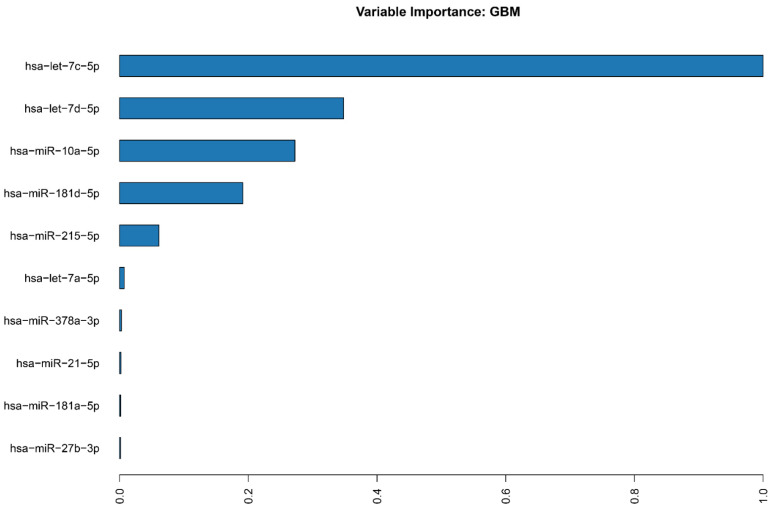
Top 10 miRNAs tagged as important variables for our GBM ML model.

From the 50 models trained by our data automatically via h2o, the one which proved to be the most accurate was a Gradient Boosting Machine (GBM_4_AutoML_7_20221005_201041). The model exhibited an AUC of 1 with a logloss of 0.0021 and a median Root-mean-square error (RMSE) of 0.046867. Unfortunately, due to the insufficient number of samples (more than 200 required, we had 95), this model defaulted to using cross-validation for its training, resulting in a more “generous” approximation of its statistics. Nonetheless, when we analyzed the importance of the variables, the top 10 of those validated the differential expression results and highlighted the mir-181 and let-7 families’ importance to our prediction model (Figure 2).

**Figure 3:**
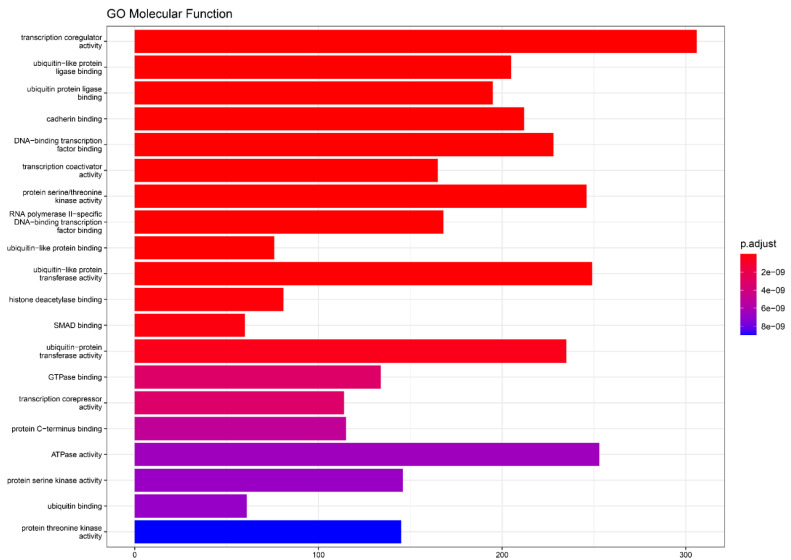
Top 20 GO:MF terms enriched by our top 10 miRNAs acting as important variables to our ML model.

Similarly to our differential expression analysis, we identified 8490 target-genes of these 10 important variables which enrich 244 GO:MF terms (Supplementary go_enrich_ML). Figure 3 depicts the top 20 GO:MF terms enriched by the top 10 miRNAs identified as important variables of our ML model. These also highlight molecular functions like “transcription coregulator activity,” “ubiquitin-like protein transferase binding,” “ubiquitin protein ligase binding,” “cadherin binding,” DNA-binding-transcription factor binding” and “protein serine/threonine kinase activity.”

## 3. Discussion

The aberrant miRNA expression and function have been previously correlated with a variety of diverse tumor types [21,22]. In fact, miRNA-coding sequences are often found within or close to cancer-associated genomic regions, resulting in the abnormal activation or inactivation of miRNAs in different cancer types [23]. Moreover, a single miRNA may have multiple targets with important differences in functions; this leads to the perception that miRNAs may exhibit opposite effects within the cell [24]. Thus, the effect of a single miRNA within a transcriptome may vary significantly, depending on the environment in which it is expressed [25]. Consequently, since the effects of miRNAs on phenotypes are context-dependent, the transcriptomes should be comparable in terms of the target-genes under consideration. For instance, a recent pan-cancer analysis concluded that miRNA behaviors are highly dependent on the complex tumor phenotypes and vice versa. The authors also suggested that members of the miR-17 and let-7 families have exhibited antagonistic behaviors in different tumor types [26]. Another pan-cancer characterization of miRNA profiles conducted by Moradi et al. identified miR-181 and miR-155, among others, to be overexpressed in the majority of cancer types included in their analysis. Of note, their work included data from the TCGA pan-cancer project, without incorporating CRC [27].

Over the last decade, the differential expression of miRNAs with regard to treatment response has been widely studied in CRC [8,28,29]. In the current work, we investigated the expression of 84 tumor-related miRNAs and used ML in order to identify miRNA signatures that seem to be associated with the response to irinotecan-based treatment in mCRC patients. Out of the top 20 miRNAs that were found to be differentially expressed in our samples, several of them have previously been linked with CRC [30,31,32]. Therefore, in line with previous observations, hsa-miR-155-5p [33,34], hsa-let-7i-5p [35], hsa-miR-92a-3p [36], hsa-miR-181d-5p [37], hsa-miR181b-5p [38], hsa-miR-10b-5p [39], hsa-let-7f-5p [40], and hsa-miR-181a-5p [41] were found to be upregulated in our cohort, whereas hsa-miR-215-5p [36,42], hsa-miR-143-3p [43,44], hsa-miR-148a-3p [45], hsa-miR-17-5p [46], hsa-miR-10a-5p [43], and hsa-let-7a-5p [47] were found to be downregulated. These miRNAs directly target critical genes in colorectal carcinogenesis and prognosis, such as PTEN [39], TUSC3 [38], PAI-1, AXIN2, CTFR [42], and predominant biological pathways such as the TGF-β signaling pathway, as well as microsatellite instability [33].

The miRNAs that were found to be dysregulated served as the basis for developing an ML classifier approach in order to detect those that could be independently related to the irinotecan-based chemotherapy response in mCRC patients. Our predictive model demonstrated that mir-181 upregulation and let-7 families’ downregulation appear to be mostly associated with non-responsiveness. Several studies have demonstrated the role of hsa-miR-181b-5p in cell proliferation, invasion and chemoresistance. For example, Li et al., reported that miR-181b-5p regulates TGF-β1-induced epithelial-to-mesenchymal transition (EMT) by targeting E-cadherin in non-small cell lung cancer stem-like cells [47]. The oncogenic potential of miR-181b in CRC was also associated with the programmed cell death 4 (PDCD4) gene, whose modulation takes place at a posttranscriptional level [48]. Similarly, the TGF-β1 signaling pathway was found to regulate the EMT of gastric cancer cells via miRNA-181b overexpression; the latest resulted in targeting the Tissue Inhibitor of Metalloproteinases 3 (TIMP3) gene through the Smad2/3/4-dependent pathway [49].

MiR-181b-5p downregulation has also been associated with the inhibition of the growth, migration and glycolysis of gallbladder cancer via the pyruvate dehydrogenase complex, under hypoxia conditions [50]. Another study found that the oncogenic role of miR-181b-5p is linked to malignancy promotion in acute lymphoblastic leukemia cells, working by decreasing the percentage of cells in the G0/G1 phase [51]. Furthermore, miR-181b downregulation has been correlated with the inhibition of colon cancer cell proliferation through the negative regulation of the NF-κB signaling pathway [52]. Moreover, miR-181b has been shown to be involved in the regulation of factors that are involved in the mitogen-activated protein kinase 1 (MAPK1) signaling pathway [53]. There are also a few studies that have investigated miR-181b in association with the response to treatment. Zhang et al. found that miR-181b-5p overexpression may play a role in sensitizing glioma cells to temozolomide by directly targeting Bcl-2 [54]. Similarly, miR-181b was found to enhance the sensitivity to gemcitabine in pancreatic ductal adenocarcinoma cells in vitro by binding to BCL-2 mRNA 3’UTR [55].

Recently, Hosseini et al. linked miR-181b downregulation to the increased sensitivity of osteosarcoma cells to cisplatin following the addition of melatonin to the treatment [56]. Consistent with this observation, our findings indicate that miR-181b-3p is overexpressed in the non-responders group, indicating that this may be attributed to drug resistance.

Hsa-miR-10b-5p has not been extensively studied in terms of the response to treatment in CRC. Schlick et al. found that there was no significant association between its expression and response to FOLFIRINOX (5-fluorouracil/leucovorin, irinotecan and oxaliplatin) in advanced pancreatic cancer patients [57]. Furthermore, another study suggested that miR-10b-5p was downregulated in triple negative breast cancer [58]. Our results indicate that its overexpression may be associated with non-responsiveness to irinotecan-based treatment in CRC patients, thus there is room for the further investigation of its role in future studies. In agreement with our findings, miR-181a-5p overexpression in CRC cell lines has already been correlated with chemoresistance [59]. More specifically, the authors proposed that the long non-coding RNA CRNDE could increase miR-181a-5p expression and regulate chemoresistance via the Wnt/β-catenin signaling pathway. Pop-Bica et al. showed that miR-181a overexpression in CRC inhibits the WIF-1 gene, resulting in the enhanced invasiveness of the malignant cells [60]. Similarly, exosomal miR-181d-5p overexpression has been associated with resistance to 5-FU by directly targeting neurocalcin δ (NCALD) [37]. Interestingly, miR-181d-5p has also been correlated with a genome instability-related competing endogenous (ceRNA) network that includes four axes; this network has been identified as a crucial modulator of the tumor microenvironment, cancer stemness and resistance to immunotherapy [61]. Regarding miR-301a-3p, our findings coincide with those that associate its upregulation with cell proliferation, invasion and migration and the inhibition of cell apoptosis in CRC [62]. Despite the fact that its role in CRC drug resistance/response has not been investigated yet, it has been reported that the overexpression of miR-301a-3p promoted gemcitabine resistance in pancreatic cancer cells, in vitro, by regulating the expression of PTEN [63]. Moreover, consistent with our results, miR-92a-3p has been linked with 5-FU/oxaliplatin resistance in CRC [64]. The authors suggested that the increased expression of miR-92a-3p activates the Wnt/β-catenin signaling pathway and suppresses mitochondrial apoptosis by directly inhibiting FBXW7 and MOAP1. There are few studies investigating the role of miR-155-5p in CRC drug resistance/response. For example, in contrast to our findings, Yin et al. found that miR-155-5p expression was frequently decreased in tumor-associated macrophages (TAMs), resulting in C/EBPβ overexpression and IL-6 activation. Subsequently, TAM-secreted IL-6 led to chemoresistance through the activation of the IL6R/STAT3/miR-204-5p pathway [65]. In line with this observation, another work suggested that the elevated expression of miR-155-5p suppresses MDR1 expression, which blocks the efflux of 5-FU, enhancing the sensitivity of CRC to 5-FU [66]. On the other hand, there are studies showing that miR-155-5p contributes to drug resistance in other cancer types [67,68]. Another miRNA that we found to be upregulated in non-responders is hsa-miR-30c-5p. Little is known about its role in cancer drug resistance. In opposition to our expectations and results, the high expression of miR-30c has been previously associated with better survival in CRC [69]. Moreover, Guo et al. suggested that circ3823 could act as a ceRNA for hsa-miR-30c-5p and reverse the restrictive effect on its target gene TCF7, which subsequently promotes CRC progression [70]. Taken together, further research is needed in order to derive valid conclusions about the role of hsa-miR-30c-5p in CRC chemoresistance.

On the other hand, let-7 family members, namely hsa-let-7c-5p, hsa-let-7d-5p, and hsa-let 7a-5p, exhibited significantly lower expression levels in non-responders compared to the control group in our study. Members of the let-7 family are known tumor suppressors [71] that modulate oncogenes such as HMGA2 [72], MYC [73] and RAS [74]. Danac et al. suggested that circPVT1 upregulates NRAS by inhibiting let-7 and thus promotes oncogenic phenotypes in CRC cells [75]. The role of let-7c-5p has not been extensively investigated in CRC. Pidikova et al. reported that let-7c-5p has been found to be downregulated in CRC compared to normal tissues, indicating its potential as a tumor suppressor [76]. However, it is known that it acts as a tumor suppressor in miRNA. Indeed, the downregulation of let-7c-5p has been linked with oncogenic potential in CRC tissues, whereas its upregulation has been associated with the inhibition of cell growth through targeting MMP11 (matrix metallopeptidase 11) and PBX3 (PBX homeobox 3 gene [77]. Its similar action has been reported in hepatocellular carcinoma, since sponging let-7c-5p has been associated with tumor proliferation and invasion [78]. In agreement with our results, let-7c-5p overexpression has been correlated with (i) a good prognosis in other gastrointestinal cancers, such as oesophageal carcinoma, as well as (ii) sensitivity to anticancer therapy [79]. Although hsa-let-7d-5p has not been studied so far in colorectal cancer drug response, its overexpression has been linked with sensitivity to tivantinib in breast cancer cell lines [80]. The authors suggested that hsa-let-7d-5p targets NKIRAS2, an inhibitor of the transcription factor NF-κB. Moreover, it is known that irinotecan activates the NF-κB pathway in order to impair chemotherapy-induced apoptosis. Therefore, the negative regulation of the NF-κB signaling pathway by hsa-let-7d-5p downregulation may be proposed as a potential mechanism of drug resistance. Similarly, hsa-let-7a-5p has not been investigated in association with CRC response so far. Genes encoding ubiquitin, such as UHRF2 (Ubiquitin Like With PHD And Ring Finger Domains 2) [81], RTKN (Rhotekin) [82] and MYC [83], have been identified as target genes for let-7a-5p. This results in cell cycle arrest and the inhibition of cell growth. Of note, our prediction model showcased that hsa-let-7d-5p resulted sixth among the 10 most differentially expressed miRNAs in non-responders to irinotecan-based therapies. Therefore, its potential role in CRC resistance could be further elucidated in future studies.

Contrary to this trend, other members of the let-7 family, such as hsa-let-7f-5p and hsa-let-7i-5p, were found upregulated in the non-responders group. Niculae et al. have also reported that both of these miRNAs were also found downregulated in 25 tumoral tissues compared with the corresponding adjacent peritumoral tissues, suggesting that they may possibly be associated with perineural invasion in CRC by targeting the insulin-like growth factor (IGF) signaling system [71]. As stated before, the horizontal hypothesis that miRNA families exhibit a common fashion in different phenotypic cancer environments has already been challenged by Dhawan et al., underlining the autonomous behavior of a single miRNA, regardless of its grouping by family [26].

Among the rest of the miRNAs that appear to be involved with non-responsiveness in our work, most of them have not been extensively investigated in CRC drug response/resistance. For example, Sakatani et al. found that the addition of melatonin resulted in enhanced 5-FU cytotoxicity; mechanistically, melatonin increased the expression of miR-215-5p, which led to thymidylate synthase gene (TYMS) downregulation [84]. Our investigation also revealed miR-215-5p downregulation in the non-responders group; given that the great majority of the administered regimens include fluoropyrimidines, we could assume that the decreased expression of miR-215-5p may have resulted in TYMS overexpression and thus chemotherapy failure [85]. Moreover, in line with previous observations, miR-143-3p downregulation has been associated with poorer responses in CRC patients [34]. In addition, the authors found that miR-10a-5p was overexpressed in non-responders, which is the opposite of our findings. This discrepancy may be attributed to the fact that the patient population in their work was receiving chemotherapy plus bevacizumab, whereas our pool of patients included several therapeutic schemes. The role of miR-142-5p has not been investigated in CRC drug resistance so far. However, similarly to our results, its overexpression has already been correlated with the promotion of chemosensitivity in other cancer types [86,87]. On the other hand, miR-148a-3p has been identified as a miRNA which promotes anti-tumoral immune responses as well as chemosensitivity in CRC cells [88]. This is in agreement with our finding that its downregulation relates to non-responsiveness. Similarly, miR-122-5p has been implicated in angiogenesis promotion, via its downregulation by a long non-coding RNA (CRAFT16) which upregulates the target oncogene FOS; subsequently, FOS overexpression upregulates Vascular Endothelial Growth Factor D (VEGFD) [89]. Finally, the last one of the top 10 significantly downregulated miRNAs in our experimental setting was miR-17-5p. In contradiction to our results, which support the observation that this miRNA is downregulated in non-responders, a few recent papers show that miR-17-5p is upregulated in many different chemo/radio-resistant cancer types, including CRC [90,91]. Of note, Despotovic et al. suggested that miR-17-5p could serve as a candidate biomarker via the regulation of the TGF-β Signaling pathway for monitoring the response to FOLFIRI in mCRC patients [46].

Interestingly, a recent integrated analysis for miRNA expression revealed miR-129-5p as a potential predictive biomarker for OS in stage IV CRC patients. However, as the authors also mentioned in their work, a solo miRNA signature is unlikely to provide an accurate, sensitive and specific prognostic tool. Besides, their analysis included a small number of patients (below 30). Furthermore, another interesting point that they addressed refers to the assumption that different miRNAs may be associated with the prognosis of a specific CRC stage [92]. In addition, Zhu et al. used bioinformatic methods to identify nine key miRNAs (namely, miR-217, miR-144, miR-129, miR-125a, miR-125b, miR-375, miR-328, miR-486, and miR-194) as potential prognostic biomarkers in CRC. However, none of the reported miRNAs were consistent with our results. It should also be noted that their findings were never associated with a prognostic value in CRC. Moreover, their analysis was based on the TCGA database, whereas our data came from an actual clinical population of mCRC patients [93].

The gene ontology analysis in our study identified molecular functions such as cadherin binding, the modulation of cell adhesion molecules (CAMs) and transcription coregulation. Indeed, our results are in accordance with the existing literature supporting the observation that the aberration of the epigenetic machinery is associated with drug resistance to conventional drugs used for CRC treatment, such as 5-fluorouracil, oxaliplatin, and irinotecan [94]. On the other hand, regarding CAMs, our results differ from those reported in the literature. For example, our results showed that cadherin under-expression may be linked to non-responsiveness to irinotecan-based treatment, whereas other authors have reported that decreased E-cadherin expression resulted in increased sensitivity to irinotecan and oxaliplatin [95,96]. Moreover, our results highlight the importance of ubiquitination, a post-translational modification which seems to be associated with non-responders. Indeed, the deregulation of anaphase-promoting complex (a specific ubiquitin ligase)-dependent proteolysis has been previously associated with the genomic instability of cancer cells [97]. Consistent with previous observations, our findings indicate that the loss of ubiquitin ligase correlates with a poor prognosis in patients receiving fluoropyrimidine-based chemotherapy. The mechanism proposed by Wang et al. suggests that the overexpression of a novel E3 ubiquitin ligase enhanced chemosensitivity to fluoropyrimidine-based chemotherapy by suppressing the activation of the c-Myc signaling pathway [98]. Furthermore, it has been proposed that an acquired resistance to irinotecan has been linked with the expression of the inhibitors of apoptosis (IAP) family members (such as c-IAP1, c-IAP2, etc.). The proposal of chemosensitivity to irinotecan was also investigated recently [99]. The authors proposed that IAP antagonists can activate E3 ubiquitin ligase activity in IAPs, which ubiquitinate themselves and regulate both the canonical and non-canonical NF-κB signaling pathways. Therefore, miRNA expression may have a direct impact on the response to treatment in mCRC patients via regulating essential pathways that are related to tumor promotion.

In the current work, we used a hybrid ML method in an attempt to provide predictive value to our results, as previously reported [14]. Differential expression (DE) analysis can accurately distinguish and characterize two conditions (in our case, responders vs. non-responders) based on inferences made using the current data, while ML is more robust, can take under consideration more factors and can provide a working model for predicting whether a patient will fall under one of the groups under investigation based on their transcriptome. Moreover, ML approaches work better when there are multiple factors to consider. Additionally, Abbas et al. provide another example of ML being more suitable for expression data. More specifically, the authors conducted an assessment combining DE analysis, supervised feature selection for the identification of the DEGs that exhibit the optimum relevance, and ML methods in order to determine the biased power of the genes under investigation [15]. Similarly, our approach leverages (i) statistical DE analysis for identifying tumor-related miRNAs using gene expression profiles from blood samples derived from 95 mCRC patients, (ii) supervised ML methods used to further assess and refine the expression results and identify the most important miRNAs that have a predictive value for response to therapy and (iii) gene ontology analysis for the identification of clinically significant DEGs and the corresponding functional pathways.

Though we conducted an extensive ML and bioinformatics analysis and confirmed the classification by cross-validation, our work has some limitations. As mentioned before, the model that we used required more than 200 samples and our sample pool was only from one oncology clinic. The route toward discovering the predictive biomarkers of drug response, especially in cases of combination therapies, requires both effective computational tools and a considerable number of samples. In addition to that, the dysregulation of miRNA expression during multi-drug treatment cannot be accurately attributed to one agent, and this may explain any inconsistencies between our results and previously published ones. Finally, cross-validation resulted in a more generous approximation of our accuracy scores. Therefore, our results need to be verified by future studies with larger datasets that can efficiently perform the treatment using different training and validation sets.

However, despite these limitations, our work included an actual clinical population, compared to other in vitro studies of CRC cell lines or in silico experiments. Furthermore, this is the first study to investigate the differential miRNA expression profiles and the response to irinotecan-based treatment in mCRC patients, based on experimental data, ML and gene ontology analysis.

## 4. Materials and Methods

### 4.1. Patients and Blood Samples

Blood samples from 95 patients with histologically confirmed mCRC were included in our study. The inclusion criteria were (1) no previous exposure to chemotherapy, (2) adequate bone marrow, renal and hepatic function and (3) performance status 0–2 (ECOG scale). All participants received irinotecan-based chemotherapy combined with fluoropyrimidines (5-FU or capecitabine) and/or biologic agents (aflibercept, bevacizumab, cetuximab or panitumumab). Chemotherapy was administered for a maximum of 12 cycles, until progressive disease or until unacceptable toxicity. Treatment regimens were in accordance with the therapeutic protocols recommended by the Hellenic Society of Medical Oncology [100]. Response to treatment was assessed using the Response Evaluation Criteria in Solid Tumors (RECIST v. 1.1) [101]. All cases were recruited in the Second Department of Medical Oncology of the General Oncology Hospital of Kifissia “Agioi Anargiroi,” Attica, Greece, between 2017 and 2021. This case-control study was conducted in accordance with the Declaration of Helsinki and approval was obtained by the Hospital’s Scientific Review Board. All study participants signed an informed consent form. Table 2 summarizes the clinicopathological data of the patients.

### 4.2. MiRNA Expression

MiRNA isolation was carried out with the NucleoSpin miRNA kit (Machnery-Nagel, Düren, Germany). 500 ng of RNA were reverse-transcribed with the miScript II RT Kit (Qiagen, Hilden, Germany) and loaded in the miScript™ miRNA PCR Array Human Cancer PathwayFinder (MIHS-102Z, Qiagen), which tests samples for the expression of 84 miRNAs. These miRNAs have been previously associated with the diagnosis and staging of various tumors. In addition to the 84 tumor-related miRNAs, the array also provides 12 housekeeping genes for normalization. Samples were grouped into 2 categories of responders and non-responders to therapeutic intervention with irinotecan-based chemotherapy in patients suffering from mCRC. The 2^−ΔCt^ method was employed to calculate individual within-sample miRNA expression, normalized using the geometric mean of all controls, while, the 2^−ΔΔCt^ approach was used to calculate the fold regulation between the groups (responders were considered the control group). For the fold regulation, FDR corrected (Benjamini-Hochberg) *p*-values (Student’s *t*-test) and 95% Confidence Interval values, were calculated.

### 4.3. Machine Learning Model

To further assess and refine the expression results and identify the most important miRNAs that have a predictive value for response to therapy, we used the h2o R package [102]. ΔCt values (Supplementary Table for_ml.csv), calculated previously for all miRNAs of the array, were provided as input for this process. The automl function of h2o allowed for the evaluation of 50 ML models to identify the one that performs better in terms of accuracy and sensitivity. Area Under the Curve (AUC), logloss and confusion matrices were used to assess the models. Deep Learning and Ensemble models were excluded from this process. All models used the same random 70–30% partitioning of the samples for training and testing accordingly.

### 4.4. miRNA Target Genes and Molecular Function Analysis

The top 10 miRNAs reported as important variables (h2o.varimp function) for the selected ML model were used as input for the multimir R package [103] to gather all genes whose transcription is known to be affected by them, using validated miRNA-target interactions from 3 databases (“mirecords” [104], “mirtarbase” [105] and “tarbase” [106]). The same process was duplicated for the top 10 upregulated and downregulated miRNAs highlighted from the differential expression analysis. Finally, the clusterprofiler package [107] was used on the genes resulting from the previous step for both approaches to identify enriched Gene Ontology Molecular Function Terms (GO-MF) [108]. The *p*-values for the GO-MF terms were calculated using one-sided Fisher’s exact test and were adjusted and sorted by FDR value. Only those of *p*.adjust (q-value) < 0.05 were considered in our analyses.

## 5. Conclusions

A machine learning approach may be accurate in predicting the response to irinotecan-based treatment in mCRC patients. Furthermore, a panel of miRNAs, with miR-181 and let families as a focus, may serve as strong predictors of non-responsiveness to treatment.

## Figures and Tables

**Table 1:** Differentially expressed miRNAs of ±2 fold regulation and *p*.adjust < 0.05 for non-responders vs. responders comparison.

miRNA	Fold Regulation	*p*-Value	*p*-Adjusted	Fold Change	95% CI
**hsa-miR-181b-5p**	116.66	0.00000	0.00000	116.66	(40.84, 192.47)
**hsa-miR-10b-5p**	47.20	0.00000	0.00000	47.20	(16.08, 78.31)
**hsa-let-7f-5p**	24.32	0.00188	0.00295	24.32	(9.28, 39.36)
**hsa-miR-181a-5p**	24.03	0.00000	0.00000	24.03	(13.52, 34.53)
**hsa-miR-181d-5p**	23.33	0.00000	0.00000	23.33	(12.72, 33.94)
**hsa-miR-301a-3p**	23.21	0.00000	0.00000	23.21	(14.58, 31.83)
**hsa-miR-92a-3p**	6.11	0.00000	0.00000	6.11	(3.00, 9.22)
**hsa-miR-155-5p**	6.11	0.00000	0.00000	6.11	(3.00, 9.22)
**hsa-miR-30c-5p**	4.06	0.00000	0.00000	4.06	(2.19, 5.94)
**hsa-let-7i-5p**	3.78	0.03537	0.04421	3.78	(1.51, 6.04)
**hsa-miR-29a-3p**	3.77	0.00009	0.00019	3.77	(2.17, 5.37)
**hsa-miR-138-5p**	3.70	0.00000	0.00000	3.70	(2.46, 4.95)
**hsa-miR-193b-3p**	3.51	0.00083	0.00143	3.51	(1.70, 5.32)
**hsa-miR-133b**	2.52	0.00010	0.00020	2.52	(0.99, 4.05)
**hsa-miR-218-5p**	2.28	0.00059	0.00112	2.28	(1.45, 3.11)
**hsa-miR-150-5p**	2.10	0.01732	0.02215	2.10	(1.29, 2.91)
**hsa-miR-196a-5p**	−2.18	0.00797	0.01124	0.46	(0.17, 0.74)
**hsa-miR-20a-5p**	−2.85	0.00004	0.00009	0.35	(0.22, 0.49)
**hsa-miR-27b-3p**	−4.10	0.00075	0.00133	0.24	(0.14, 0.35)
**hsa-miR-200c-3p**	−4.28	0.00000	0.00000	0.23	(0.14, 0.32)
**hsa-miR-193a-5p**	−6.74	0.00000	0.00000	0.15	(0.10, 0.20)
**hsa-miR-134-5p**	−7.02	0.00000	0.00000	0.14	(0.09, 0.20)
**hsa-miR-20b-5p**	−7.34	0.00000	0.00000	0.14	(0.07, 0.20)
**hsa-miR-124-3p**	−7.56	0.00000	0.00000	0.13	(0.09, 0.18)
**hsa-miR-17-5p**	−8.35	0.00000	0.00000	0.12	(0.07, 0.17)
**hsa-miR-122-5p**	−8.71	0.00000	0.00000	0.11	(0.06, 0.17)
**hsa-miR-148a-3p**	−14.62	0.00000	0.00000	0.07	(0.03, 0.11)
**hsa-miR-142-5p**	−16.53	0.00001	0.00003	0.06	(0.03, 0.09)
**hsa-miR-10a-5p**	−17.65	0.00006	0.00013	0.06	(0.04, 0.08)
**hsa-let-7a-5p**	−19.54	0.00000	0.00000	0.05	(0.03, 0.07)
**hsa-miR-143-3p**	−23.44	0.00001	0.00003	0.04	(0.02, 0.07)
**hsa-miR-215-5p**	−26.06	0.00002	0.00005	0.04	(0.02, 0.05)
**hsa-let-7c-5p**	−36.94	0.00000	0.00000	0.03	(0.01, 0.04)
**hsa-let-7d-5p**	−54.23	0.00000	0.00000	0.02	(0.01, 0.03)

**Table 2:** Clinicopathological characteristics of the study population.

Characteristics		Number of Patients
Gender	Male	58
	Female	37
Surgery on primary	Yes	83
	No	12
Primary Location	Cecum	9
	Ascending colon	5
	Ascending/Transverse colon	1
	Transverse colon	3
	Splenic Flexure	3
	Descending colon	3
	Descending colon/Rectum	1
	Sigmoid	26
	Rectosigmoid	21
	Rectum	23
Metastasis site	Lung	23
	Liver	37
	Peritoneum	7
	Adrenal gland	1
	Multiple sites	20
	Local Recurrence	7
Therapeutics	Panitumumab, irinotecan and 5-FU	10
	Panitumumab, irinotecan and capecitabine	8
	Bevacizumab, irinotecan and 5-FU	21
	Bevacizumab, irinotecan and capecitabine	27
	Aflibercept, irinotecan and 5-FU	3
	Cetuximab, irinotecan and 5-FU	4
	Bevacizumab, irinotecan, 5-FU and oxaliplatin	1
	Bevacizumab, irinotecan, capecitabine and oxaliplatin	1
	Bevacizumab, irinotecan and oxaliplatin	1
	Capecitabine and irinotecan	10
	Oxaliplatin and irinotecan	3
	Irinotecan and 5-FU	5
	Panitumumab and irinotecan	1
Response status	Stable Disease (SD)	47
	Progressive Disease (PD)	48

## Data Availability

Not applicable.

## Supplementary Materials

The following supporting information can be downloaded at: https://www.mdpi.com/article/10.3390/ijms24010046/s1.
